# Bilateral periorbital ecchymosis in a case with dengue fever

**DOI:** 10.4103/0301-4738.49407

**Published:** 2009

**Authors:** Vinod Kumar, Basudeb Ghosh, Usha K Raina, Neha Goel

**Affiliations:** Guru Nanak Eye Centre, Maulana Azad Medical College, New Delhi - 110 002, India

Dear Editor,

Dengue is caused by one of four closely related, but antigenically distinct, virus serotypes of the genus *Flavivirus* and spreads by *Aedes aegypti*, a domestic, day-biting mosquito that prefers to feed on humans.[1] Infections produce a spectrum of clinical illness ranging from a nonspecific viral syndrome to severe and fatal hemorrhagic disease resulting from increased vascular permeability and decreased platelet count.[1] Varying ocular findings in dengue, though rare are known,[2,4] petechial hemorrhages in conjunctiva being the commonest.[2] Maculopathy is another common manifestation.[5] We hereby, report a case of bilateral periorbital ecchymosis in a case of dengue hemorrhagic fever.

**Figure 1:**
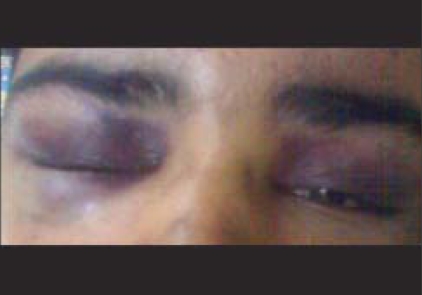
Bilateral periorbital ecchymosis in a patient with dengue fever

A 22-year-old male patient admitted with the diagnosis of dengue hemorrhagic fever was referred from the medicine department of our hospital with complaints of bilateral periorbital swelling and bruising of one-day duration. There was no history of any blunt trauma to the orbit or the head. On examination, both the eyes had periorbital ecchymosis significantly worse in the right eye [Fig. 1]. Both the ocular globes were normal with no restriction of motility and a visual acuity of 20/20 in both eyes. Pupils were reactive to light in both eyes with no relative afferent pupillary defect. Intraocular pressure was 18 mm Hg in both eyes as measured by applanation tonometry. A dilated fundus examination revealed normal fundus with no evidence of hemorrhages or exudates. There was no evidence of ecchymosis elsewhere in the body. Complete blood counts of patient showed a significantly reduced platelet count of 16000/μL (normal range 150000 – 400000/μL). The diagnosis of dengue fever was confirmed by the detection of dengue IgM antibodies.

The patient received six units of platelet concentrate and supportive therapy was given in the form of antipyretics and volume replacement. No specific treatment was added from the ophthalmic point of view. He was followed up after one week, and there was no change in the periorbital ecchymosis. Platelet count was 28000/μL at this point. Subsequently the patient was lost to follow-up.

Dengue fever is considered to be rarely associated with ocular manifestations. The onset of symptoms appears to coincide with the resolution of fever and the nadir of thrombocytopenia.[6]

Reported ocular manifestations of dengue fever include subconjunctival hemorrhage, vitreous hemorrhage, choroidal effusions, relative central scotoma, intraretinal hemorrhages, Roth spots, cotton-wool spots, retinal edema, blurring of the optic disk and maculopathy. Platelet count less than 50000/μL predisposes to ocular hemorrhages.[2] Nevertheless, periorbital ecchymosis has not been described as part of the clinical spectrum of dengue fever or any other viral fever. As it occurred with a significantly low platelet count, it appears to be related to thrombocytopenia.

Patients with dengue fever who report visual symptoms should be evaluated promptly. Although there is no specific therapy, retinal hemorrhage may be an indication for early and aggressive correction of thrombocytopenia. Ocular alterations in dengue are usually self-limiting. Most of the findings resolve without specific treatment, but occasionally visual recovery may be prolonged or vision may remain permanently impaired in patients with a severe maculopathy.[4] To the best of our knowledge this is the first reported case of bilateral periorbital ecchymosis in a case of dengue hemorrhagic fever.
